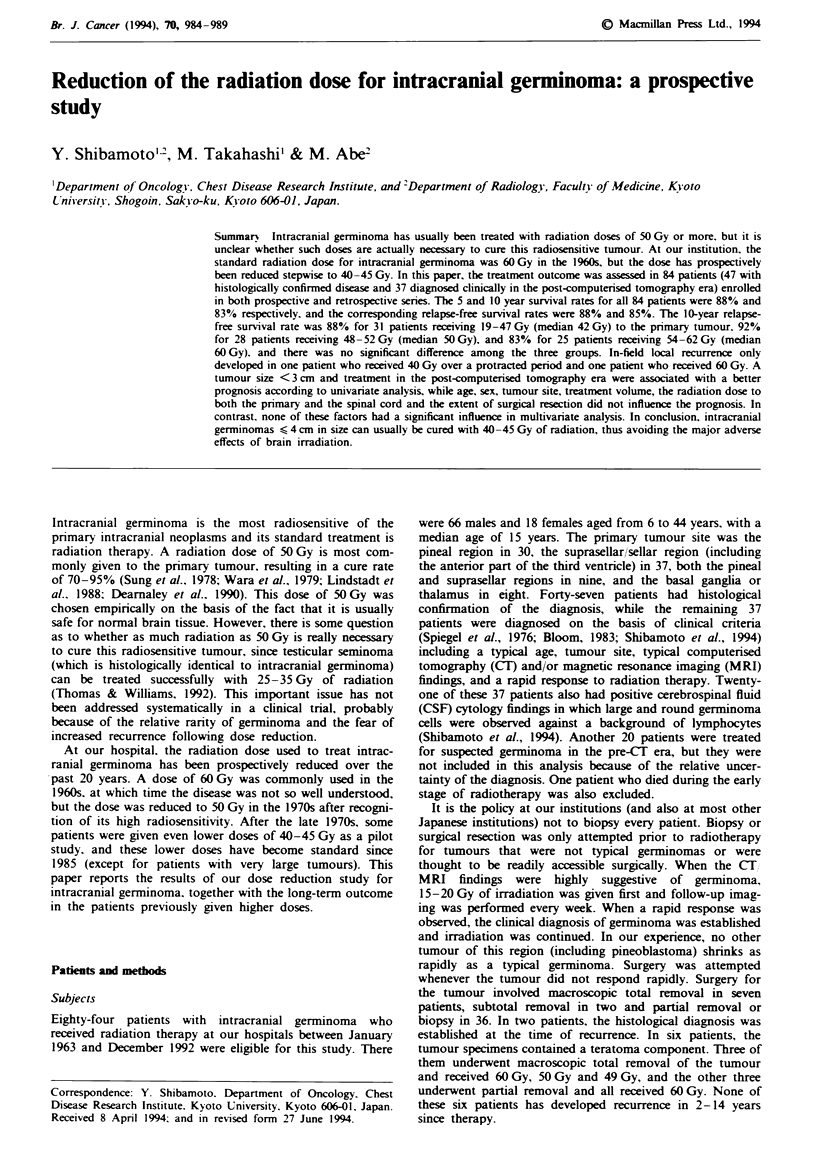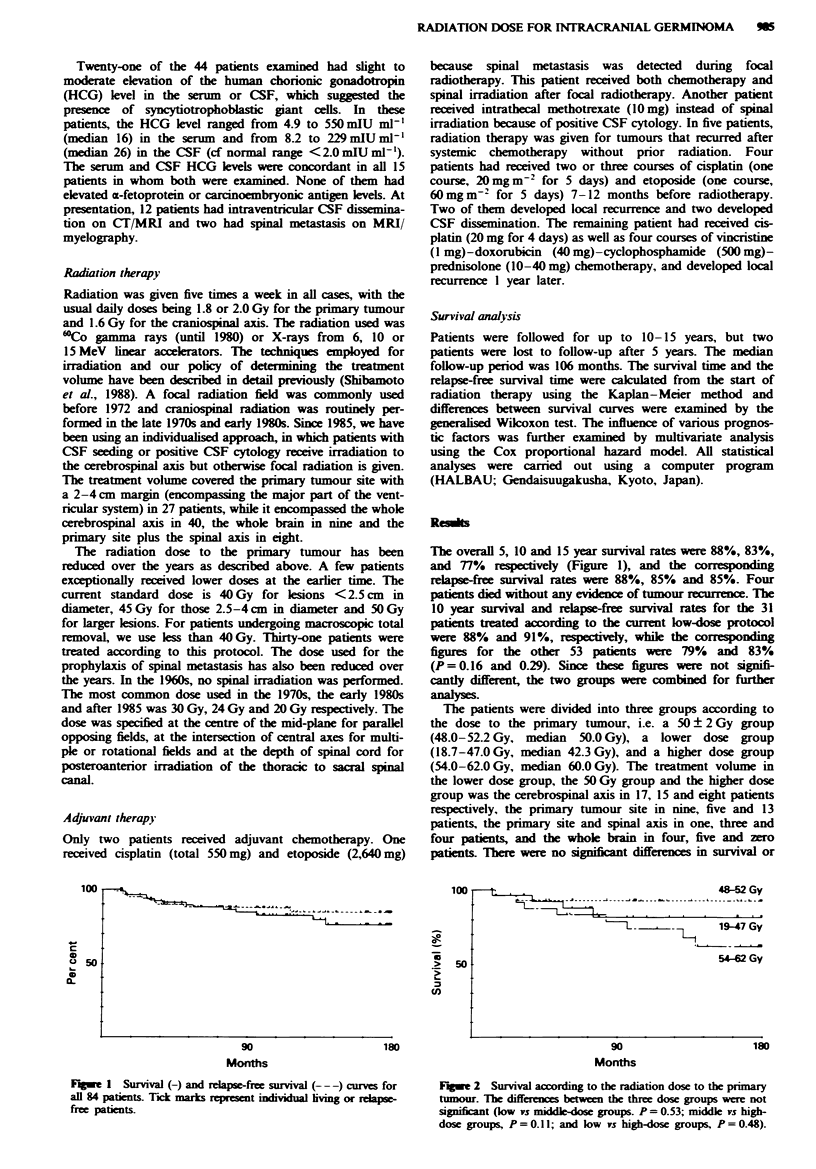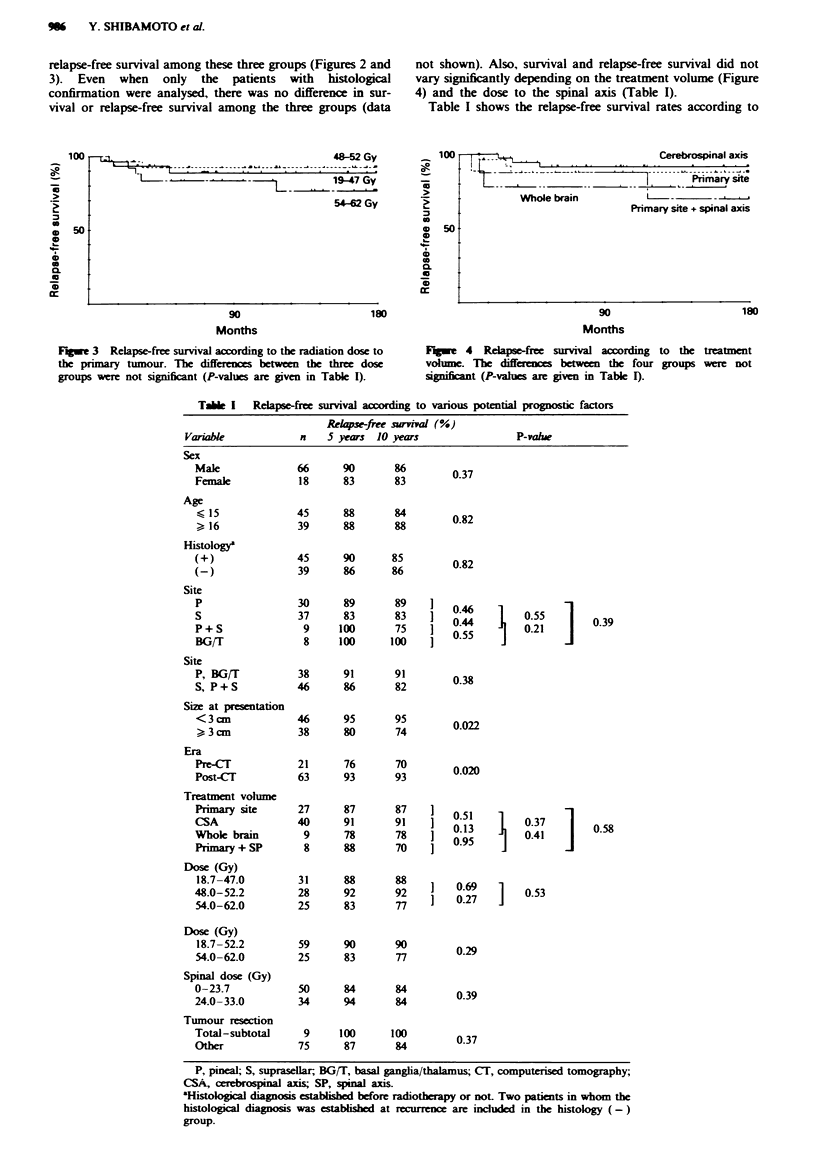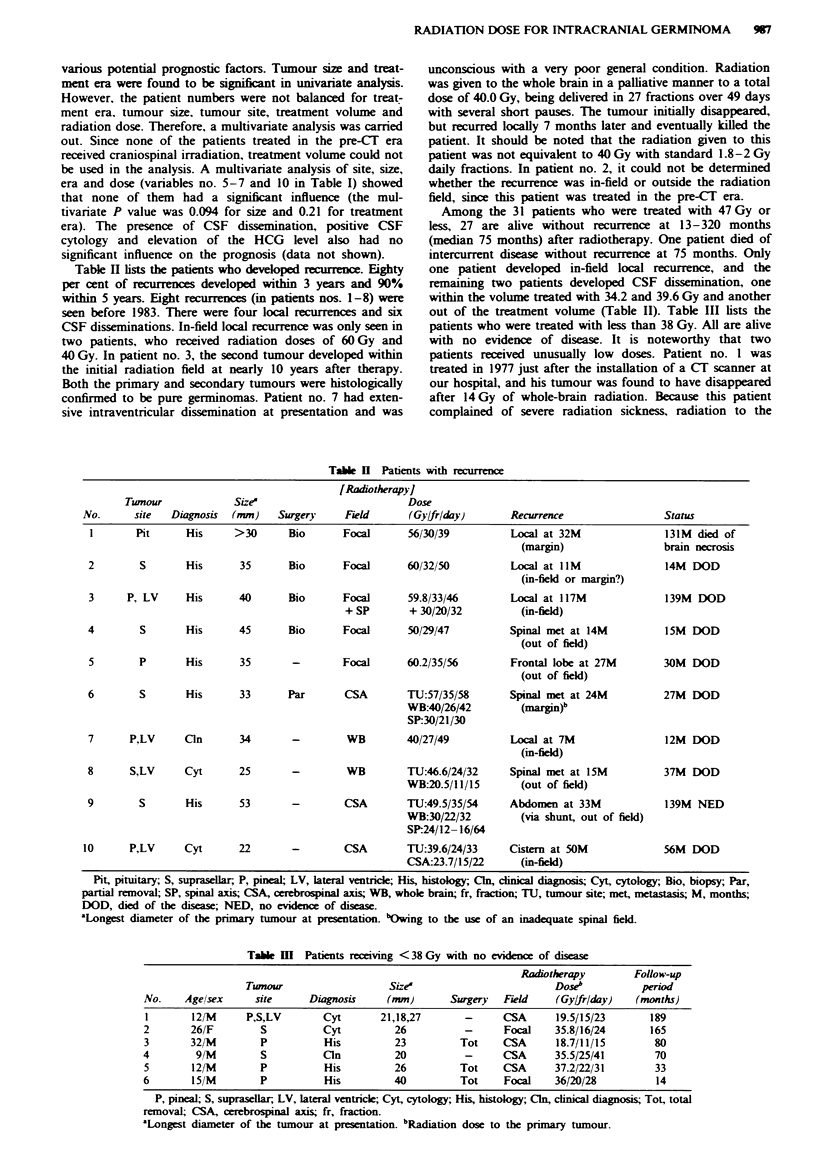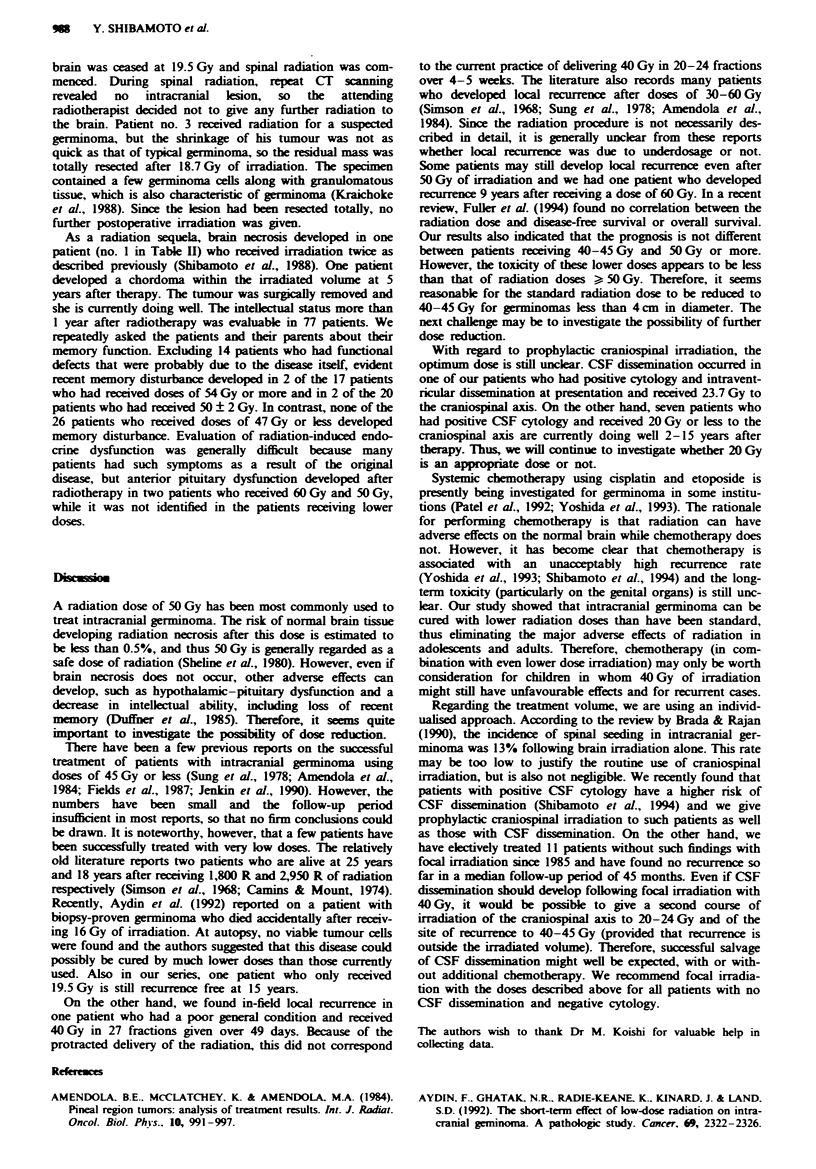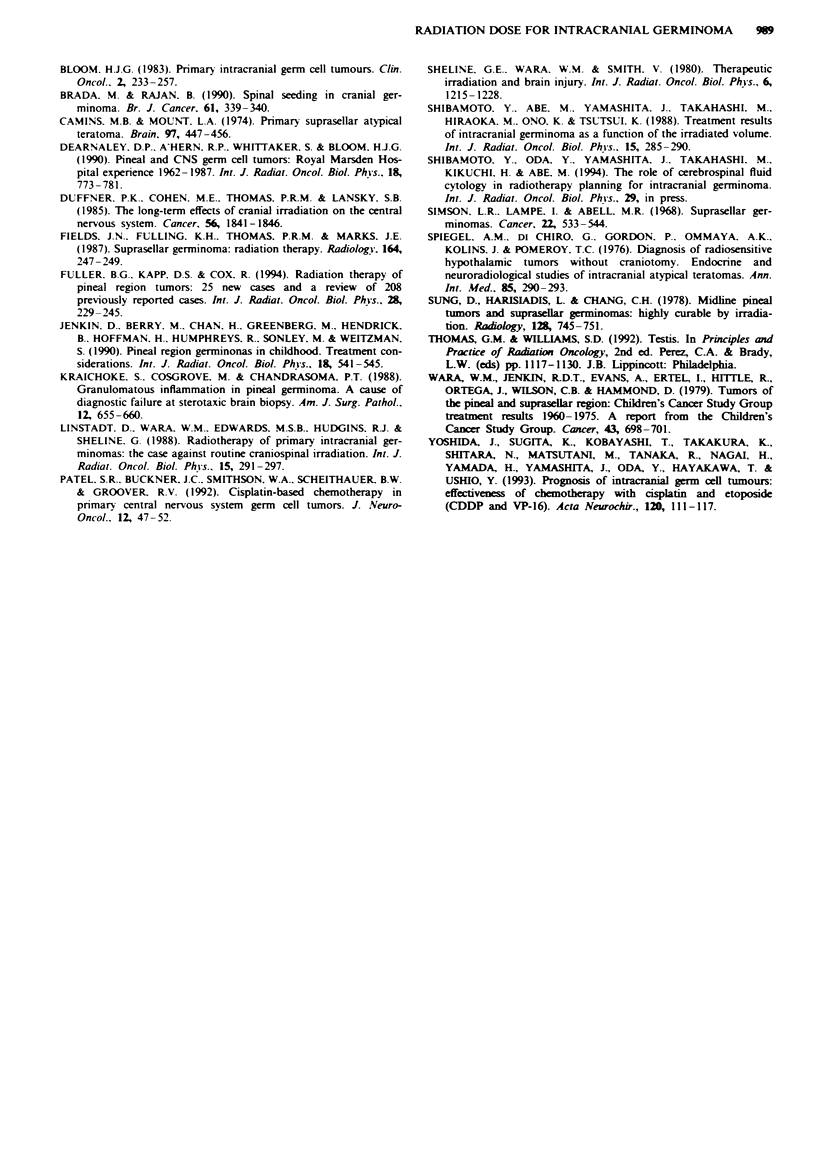# Reduction of the radiation dose for intracranial germinoma: a prospective study.

**DOI:** 10.1038/bjc.1994.434

**Published:** 1994-11

**Authors:** Y. Shibamoto, M. Takahashi, M. Abe

**Affiliations:** Department of Oncology, Faculty of Medicine, Kyoto University, Japan.

## Abstract

Intracranial germinoma has usually been treated with radiation doses of 50 Gy or more, but it is unclear whether such doses are actually necessary to cure this radiosensitive tumour. At our institution, the standard radiation dose for intracranial germinoma was 60 Gy in the 1960s, but the dose has prospectively been reduced stepwise to 40-45 Gy. In this paper, the treatment outcome was assessed in 84 patients (47 with histologically confirmed disease and 37 diagnosed clinically in the post-computerised tomography era) enrolled in both prospective and retrospective series. The 5 and 10 years survival rates for all 84 patients were 88% and 83% respectively, and the corresponding relapse-free survival rates were 88% and 85%. The 10-year relapse-free survival rate was 88% for 31 patients receiving 19-47 Gy (median 42 Gy) to the primary tumour, 92% for 28 patients receiving 48-52 Gy (median 50 Gy), and 83% for 25 patients receiving 54-62 Gy (median 60 Gy), and there was no significant difference among the three groups. In-field local recurrence only developed in one patient who received 40 Gy over a protracted period and one patient who received 60 Gy. A tumour size < 3 cm and treatment in the post-computerised tomography era were associated with a better prognosis according to univariate analysis, while age, sex, tumour site, treatment volume, the radiation dose to both the primary and the spinal cord and the extent of surgical resection did not influence the prognosis. In contrast, none of these factors had a significant influence in multivariate analysis. In conclusion, intracranial germinomas < or = 4 cm in size can usually be cured with 40-45 Gy of radiation, thus avoiding the major adverse effects of brain irradiation.


Br. J. Cancer (1994), 70, 984-989 © Macmillan Press Ltd., 1994

### Reduction of the radiation dose for intracranial germinoma: a prospective study

Y. Shibamoto[1,2], M. Takahashi[1] & M. Abe[2]

[1]*Department of Oncology, Chest Disease Research Institute, and* [2]*Department of Radiology, Faculty of Medicine, Kyoto University, Shogoin, Sakyo-ku, Kyoto 606-01, Japan.*

**Summary** Intracranial germinoma has usually been treated with radiation doses of 50 Gy or more, but it is unclear whether such doses are actually necessary to cure this radiosensitive tumour. At our institution, the standard radiation dose for intracranial germinoma was 60 Gy in the 1960s, but the dose has prospectively been reduced stepwise to 40-45 Gy. In this paper, the treatment outcome was assessed in 84 patients (47 with histologically confirmed disease and 37 diagnosed clinically in the post-computerised tomography era) enrolled in both prospective and retrospective series. The 5 and 10 year survival rates for all 84 patients were 88% and 83% respectively, and the corresponding relapse-free survival rates were 88% and 85%. The 10-year relapse-free survival rate was 88% for 31 patients receiving 19-47 Gy (median 42 Gy) to the primary tumour, 92% for 28 patients receiving 48-52 Gy (median 50 Gy), and 83% for 25 patients receiving 54-62 Gy (median 60 Gy), and there was no significant difference among the three groups. In-field local recurrence only developed in one patient who received 40 Gy over a protracted period and one patient who received 60 Gy. A tumour size <3 cm and treatment in the post-computerised tomography era were associated with a better prognosis according to univariate analysis, while age, sex, tumour site, treatment volume, the radiation dose to both the primary and the spinal cord and the extent of surgical resection did not influence the prognosis. In contrast, none of these factors had a significant influence in multivariate analysis. In conclusion, intracranial germinomas ≤ 4 cm in size can usually be cured with 40-45 Gy of radiation, thus avoiding the major adverse effects of brain irradiation.

Intracranial germinoma is the most radiosensitive of the primary intracranial neoplasms and its standard treatment is radiation therapy. A radiation dose of 50 Gy is most commonly given to the primary tumour, resulting in a cure rate of 70-95% (Sung *et al.*, 1978; Wara *et al.*, 1979; Lindstadt *et al.*, 1988; Dearnaley *et al.*, 1990). This dose of 50 Gy was chosen empirically on the basis of the fact that it is usually safe for normal brain tissue. However, there is some question as to whether as much radiation as 50 Gy is really necessary to cure this radiosensitive tumour, since testicular seminoma (which is histologically identical to intracranial germinoma) can be treated successfully with 25-35 Gy of radiation (Thomas & Williams, 1992). This important issue has not been addressed systematically in a clinical trial, probably because of the relative rarity of germinoma and the fear of increased recurrence following dose reduction.

At our hospital, the radiation dose used to treat intracranial germinoma has been prospectively reduced over the past 20 years. A dose of 60 Gy was commonly used in the 1960s, at which time the disease was not so well understood, but the dose was reduced to 50 Gy in the 1970s after recognition of its high radiosensitivity. After the late 1970s, some patients were given even lower doses of 40-45 Gy as a pilot study, and these lower doses have become standard since 1985 (except for patients with very large tumours). This paper reports the results of our dose reduction study for intracranial germinoma, together with the long-term outcome in the patients previously given higher doses.

### Patients and methods

### *Subjects*

Eighty-four patients with intracranial germinoma who received radiation therapy at our hospitals between January 1963 and December 1992 were eligible for this study. There were 66 males and 18 females aged from 6 to 44 years, with a median age of 15 years. The primary tumour site was the pineal region in 30, the suprasellar/sellar region (including the anterior part of the third ventricle) in 37, both the pineal and suprasellar regions in nine, and the basal ganglia or thalamus in eight. Forty-seven patients had histological confirmation of the diagnosis, while the remaining 37 patients were diagnosed on the basis of clinical criteria (Spiegel *et al.*, 1976; Bloom, 1983; Shibamoto *et al.*, 1994) including a typical age, tumour site, typical computerised tomography (CT) and/or magnetic resonance imaging (MRI) findings, and a rapid response to radiation therapy. Twenty-one of these 37 patients also had positive cerebrospinal fluid (CSF) cytology findings in which large and round germinoma cells were observed against a background of lymphocytes (Shibamoto *et al.*, 1994). Another 20 patients were treated for suspected germinoma in the pre-CT era, but they were not included in this analysis because of the relative uncertainty of the diagnosis. One patient who died during the early stage of radiotherapy was also excluded.

It is the policy at our institutions (and also at most other Japanese institutions) not to biopsy every patient. Biopsy or surgical resection was only attempted prior to radiotherapy for tumours that were not typical germinomas or were thought to be readily accessible surgically. When the CT/MRI findings were highly suggestive of germinoma, 15-20 Gy of irradiation was given first and follow-up imaging was performed every week. When a rapid response was observed, the clinical diagnosis of germinoma was established and irradiation was continued. In our experience, no other tumour of this region (including pineoblastoma) shrinks as rapidly as a typical germinoma. Surgery was attempted whenever the tumour did not respond rapidly. Surgery for the tumour involved macroscopic total removal in seven patients, subtotal removal in two and partial removal or biopsy in 36. In two patients, the histological diagnosis was established at the time of recurrence. In six patients, the tumour specimens contained a teratoma component. Three of them underwent macroscopic total removal of the tumour and received 60 Gy, 50 Gy and 49 Gy, and the other three underwent partial removal and all received 60 Gy. None of these six patients has developed recurrence in 2-14 years since therapy.

Correspondence: Y. Shibamoto, Department of Oncology, Chest Disease Research Institute, Kyoto University, Kyoto 606-01, Japan.
Received 8 April 1994; and in revised form 27 June 1994.

Twenty-one of the 44 patients examined had slight to moderate elevation of the human chorionic gonadotropin (HCG) level in the serum or CSF, which suggested the presence of syncytiotrophoblastic giant cells. In these patients, the HCG level ranged from 4.9 to 550 mIU $ml^{-1}$ (median 16) in the serum and from 8.2 to 229 mIU $ml^{-1}$ (median 26) in the CSF (cf normal range $<2.0$ mIU $ml^{-1}$). The serum and CSF HCG levels were concordant in all 15 patients in whom both were examined. None of them had elevated α-fetoprotein or carcinoembryonic antigen levels. At presentation, 12 patients had intraventricular CSF dissemination on CT/MRI and two had spinal metastasis on MRI/myelography.

*Radiation therapy*

Radiation was given five times a week in all cases, with the usual daily doses being 1.8 or 2.0 Gy for the primary tumour and 1.6 Gy for the craniospinal axis. The radiation used was $^{60}Co$ gamma rays (until 1980) or X-rays from 6, 10 or 15 MeV linear accelerators. The techniques employed for irradiation and our policy of determining the treatment volume have been described in detail previously (Shibamoto *et al.*, 1988). A focal radiation field was commonly used before 1972 and craniospinal radiation was routinely performed in the late 1970s and early 1980s. Since 1985, we have been using an individualised approach, in which patients with CSF seeding or positive CSF cytology receive irradiation to the cerebrospinal axis but otherwise focal radiation is given. The treatment volume covered the primary tumour site with a 2–4 cm margin (encompassing the major part of the ventricular system) in 27 patients, while it encompassed the whole cerebrospinal axis in 40, the whole brain in nine and the primary site plus the spinal axis in eight.

The radiation dose to the primary tumour has been reduced over the years as described above. A few patients exceptionally received lower doses at the earlier time. The current standard dose is 40 Gy for lesions $<2.5$ cm in diameter, 45 Gy for those 2.5–4 cm in diameter and 50 Gy for larger lesions. For patients undergoing macroscopic total removal, we use less than 40 Gy. Thirty-one patients were treated according to this protocol. The dose used for the prophylaxis of spinal metastasis has also been reduced over the years. In the 1960s, no spinal irradiation was performed. The most common dose used in the 1970s, the early 1980s and after 1985 was 30 Gy, 24 Gy and 20 Gy respectively. The dose was specified at the centre of the mid-plane for parallel opposing fields, at the intersection of central axes for multiple or rotational fields and at the depth of spinal cord for posteroanterior irradiation of the thoracic to sacral spinal canal.

*Adjuvant therapy*

Only two patients received adjuvant chemotherapy. One received cisplatin (total 550 mg) and etoposide (2,640 mg) because spinal metastasis was detected during focal radiotherapy. This patient received both chemotherapy and spinal irradiation after focal radiotherapy. Another patient received intrathecal methotrexate (10 mg) instead of spinal irradiation because of positive CSF cytology. In five patients, radiation therapy was given for tumours that recurred after systemic chemotherapy without prior radiation. Four patients had received two or three courses of cisplatin (one course, 20 mg $m^{-2}$ for 5 days) and etoposide (one course, 60 mg $m^{-2}$ for 5 days) 7–12 months before radiotherapy. Two of them developed local recurrence and two developed CSF dissemination. The remaining patient had received cisplatin (20 mg for 4 days) as well as four courses of vincristine (1 mg)-doxorubicin (40 mg)-cyclophosphamide (500 mg)-prednisolone (10–40 mg) chemotherapy, and developed local recurrence 1 year later.

*Survival analysis*

Patients were followed for up to 10–15 years, but two patients were lost to follow-up after 5 years. The median follow-up period was 106 months. The survival time and the relapse-free survival time were calculated from the start of radiation therapy using the Kaplan–Meier method and differences between survival curves were examined by the generalised Wilcoxon test. The influence of various prognostic factors was further examined by multivariate analysis using the Cox proportional hazard model. All statistical analyses were carried out using a computer program (HALBAU; Gendaisuugakusha, Kyoto, Japan).

### Results

The overall 5, 10 and 15 year survival rates were 88%, 83%, and 77% respectively (Figure 1), and the corresponding relapse-free survival rates were 88%, 85% and 85%. Four patients died without any evidence of tumour recurrence. The 10 year survival and relapse-free survival rates for the 31 patients treated according to the current low-dose protocol were 88% and 91%, respectively, while the corresponding figures for the other 53 patients were 79% and 83% ($P = 0.16$ and 0.29). Since these figures were not significantly different, the two groups were combined for further analyses.

The patients were divided into three groups according to the dose to the primary tumour, i.e. a $50 \pm 2$ Gy group (48.0–52.2 Gy, median 50.0 Gy), a lower dose group (18.7–47.0 Gy, median 42.3 Gy), and a higher dose group (54.0–62.0 Gy, median 60.0 Gy). The treatment volume in the lower dose group, the 50 Gy group and the higher dose group was the cerebrospinal axis in 17, 15 and eight patients respectively, the primary tumour site in nine, five and 13 patients, the primary site and spinal axis in one, three and four patients, and the whole brain in four, five and zero patients. There were no significant differences in survival or

**Figure 1** Survival (–) and relapse-free survival (- - -) curves for all 84 patients. Tick marks represent individual living or relapse-free patients.

**Figure 2** Survival according to the radiation dose to the primary tumour. The differences between the three dose groups were not significant (low *vs* middle-dose groups, $P = 0.53$; middle *vs* high-dose groups, $P = 0.11$; and low *vs* high-dose groups, $P = 0.48$).

relapse-free survival among these three groups (Figures 2 and 3). Even when only the patients with histological confirmation were analysed, there was no difference in survival or relapse-free survival among the three groups (data not shown). Also, survival and relapse-free survival did not vary significantly depending on the treatment volume (Figure 4) and the dose to the spinal axis (Table I).

Table I shows the relapse-free survival rates according to

**Figure 3** Relapse-free survival according to the radiation dose to the primary tumour. The differences between the three dose groups were not significant (*P*-values are given in Table I).

**Figure 4** Relapse-free survival according to the treatment volume. The differences between the four groups were not significant (*P*-values are given in Table I).

**Table I** Relapse-free survival according to various potential prognostic factors

| Variable | n | Relapse-free survival (%) 5 years | 10 years | P-value | | |
|---|---|---|---|---|---|---|
| Sex | | | | | | |
| Male | 66 | 90 | 86 | 0.37 | | |
| Female | 18 | 83 | 83 | | | |
| Age | | | | | | |
| ≤ 15 | 45 | 88 | 84 | 0.82 | | |
| ≥ 16 | 39 | 88 | 88 | | | |
| Histology[a] | | | | | | |
| (+) | 45 | 90 | 85 | 0.82 | | |
| (−) | 39 | 86 | 86 | | | |
| Site | | | | | | |
| P | 30 | 89 | 89 | 0.46 | | |
| S | 37 | 83 | 83 | 0.44 | 0.55 | 0.39 |
| P + S | 9 | 100 | 75 | 0.55 | 0.21 | |
| BG/T | 8 | 100 | 100 | | | |
| Site | | | | | | |
| P, BG/T | 38 | 91 | 91 | 0.38 | | |
| S, P + S | 46 | 86 | 82 | | | |
| Size at presentation | | | | | | |
| < 3 cm | 46 | 95 | 95 | 0.022 | | |
| ≥ 3 cm | 38 | 80 | 74 | | | |
| Era | | | | | | |
| Pre-CT | 21 | 76 | 70 | 0.020 | | |
| Post-CT | 63 | 93 | 93 | | | |
| Treatment volume | | | | | | |
| Primary site | 27 | 87 | 87 | 0.51 | | |
| CSA | 40 | 91 | 91 | 0.13 | 0.37 | 0.58 |
| Whole brain | 9 | 78 | 78 | 0.95 | 0.41 | |
| Primary + SP | 8 | 88 | 70 | | | |
| Dose (Gy) | | | | | | |
| 18.7–47.0 | 31 | 88 | 88 | 0.69 | 0.53 | |
| 48.0–52.2 | 28 | 92 | 92 | 0.27 | | |
| 54.0–62.0 | 25 | 83 | 77 | | | |
| Dose (Gy) | | | | | | |
| 18.7–52.2 | 59 | 90 | 90 | 0.29 | | |
| 54.0–62.0 | 25 | 83 | 77 | | | |
| Spinal dose (Gy) | | | | | | |
| 0–23.7 | 50 | 84 | 84 | 0.39 | | |
| 24.0–33.0 | 34 | 94 | 84 | | | |
| Tumour resection | | | | | | |
| Total–subtotal | 9 | 100 | 100 | 0.37 | | |
| Other | 75 | 87 | 84 | | | |

P, pineal; S, suprasellar; BG/T, basal ganglia/thalamus; CT, computerised tomography; CSA, cerebrospinal axis; SP, spinal axis.

[a]Histological diagnosis established before radiotherapy or not. Two patients in whom the histological diagnosis was established at recurrence are included in the histology ( − ) group.

various potential prognostic factors. Tumour size and treatment era were found to be significant in univariate analysis. However, the patient numbers were not balanced for treatment era, tumour size, tumour site, treatment volume and radiation dose. Therefore, a multivariate analysis was carried out. Since none of the patients treated in the pre-CT era received craniospinal irradiation, treatment volume could not be used in the analysis. A multivariate analysis of site, size, era and dose (variables no. 5–7 and 10 in Table I) showed that none of them had a significant influence (the multivariate $P$ value was 0.094 for size and 0.21 for treatment era). The presence of CSF dissemination, positive CSF cytology and elevation of the HCG level also had no significant influence on the prognosis (data not shown).

Table II lists the patients who developed recurrence. Eighty per cent of recurrences developed within 3 years and 90% within 5 years. Eight recurrences (in patients nos. 1–8) were seen before 1983. There were four local recurrences and six CSF disseminations. In-field local recurrence was only seen in two patients, who received radiation doses of 60 Gy and 40 Gy. In patient no. 3, the second tumour developed within the initial radiation field at nearly 10 years after therapy. Both the primary and secondary tumours were histologically confirmed to be pure germinomas. Patient no. 7 had extensive intraventricular dissemination at presentation and was unconscious with a very poor general condition. Radiation was given to the whole brain in a palliative manner to a total dose of 40.0 Gy, being delivered in 27 fractions over 49 days with several short pauses. The tumour initially disappeared, but recurred locally 7 months later and eventually killed the patient. It should be noted that the radiation given to this patient was not equivalent to 40 Gy with standard 1.8–2 Gy daily fractions. In patient no. 2, it could not be determined whether the recurrence was in-field or outside the radiation field, since this patient was treated in the pre-CT era.

Among the 31 patients who were treated with 47 Gy or less, 27 are alive without recurrence at 13–320 months (median 75 months) after radiotherapy. One patient died of intercurrent disease without recurrence at 75 months. Only one patient developed in-field local recurrence, and the remaining two patients developed CSF dissemination, one within the volume treated with 34.2 and 39.6 Gy and another out of the treatment volume (Table II). Table III lists the patients who were treated with less than 38 Gy. All are alive with no evidence of disease. It is noteworthy that two patients received unusually low doses. Patient no. 1 was treated in 1977 just after the installation of a CT scanner at our hospital, and his tumour was found to have disappeared after 14 Gy of whole-brain radiation. Because this patient complained of severe radiation sickness, radiation to the

**Table II** Patients with recurrence

| No. | Tumour site | Diagnosis | Size[a] (mm) | Surgery | [Radiotherapy] Field | Dose (Gy/fr/day) | Recurrence | Status |
|---|---|---|---|---|---|---|---|---|
| 1 | Pit | His | >30 | Bio | Focal | 56/30/39 | Local at 32M (margin) | 131M died of brain necrosis |
| 2 | S | His | 35 | Bio | Focal | 60/32/50 | Local at 11M (in-field or margin?) | 14M DOD |
| 3 | P, LV | His | 40 | Bio | Focal + SP | 59.8/33/46 + 30/20/32 | Local at 117M (in-field) | 139M DOD |
| 4 | S | His | 45 | Bio | Focal | 50/29/47 | Spinal met at 14M (out of field) | 15M DOD |
| 5 | P | His | 35 | – | Focal | 60.2/35/56 | Frontal lobe at 27M (out of field) | 30M DOD |
| 6 | S | His | 33 | Par | CSA | TU:57/35/58 WB:40/26/42 SP:30/21/30 | Spinal met at 24M (margin)[b] | 27M DOD |
| 7 | P,LV | Cln | 34 | – | WB | 40/27/49 | Local at 7M (in-field) | 12M DOD |
| 8 | S,LV | Cyt | 25 | – | WB | TU:46.6/24/32 WB:20.5/11/15 | Spinal met at 15M (out of field) | 37M DOD |
| 9 | S | His | 53 | – | CSA | TU:49.5/35/54 WB:30/22/32 SP:24/12-16/64 | Abdomen at 33M (via shunt, out of field) | 139M NED |
| 10 | P,LV | Cyt | 22 | – | CSA | TU:39.6/24/33 CSA:23.7/15/22 | Cistern at 50M (in-field) | 56M DOD |

Pit, pituitary; S, suprasellar; P, pineal; LV, lateral ventricle; His, histology; Cln, clinical diagnosis; Cyt, cytology; Bio, biopsy; Par, partial removal; SP, spinal axis; CSA, cerebrospinal axis; WB, whole brain; fr, fraction; TU, tumour site; met, metastasis; M, months; DOD, died of the disease; NED, no evidence of disease.
[a]Longest diameter of the primary tumour at presentation. [b]Owing to the use of an inadequate spinal field.

**Table III** Patients receiving <38 Gy with no evidence of disease

| No. | Age/sex | Tumour site | Diagnosis | Size[a] (mm) | Surgery | Radiotherapy Field | Dose[b] (Gy/fr/day) | Follow-up period (months) |
|---|---|---|---|---|---|---|---|---|
| 1 | 12/M | P,S,LV | Cyt | 21,18,27 | – | CSA | 19.5/15/23 | 189 |
| 2 | 26/F | S | Cyt | 26 | – | Focal | 35.8/16/24 | 165 |
| 3 | 32/M | P | His | 23 | Tot | CSA | 18.7/11/15 | 80 |
| 4 | 9/M | S | Cln | 20 | – | CSA | 35.5/25/41 | 70 |
| 5 | 12/M | P | His | 26 | Tot | CSA | 37.2/22/31 | 33 |
| 6 | 15/M | P | His | 40 | Tot | Focal | 36/20/28 | 14 |

P, pineal; S, suprasellar; LV, lateral ventricle; Cyt, cytology; His, histology; Cln, clinical diagnosis; Tot, total removal; CSA, cerebrospinal axis; fr, fraction.
[a]Longest diameter of the tumour at presentation. [b]Radiation dose to the primary tumour.

brain was ceased at 19.5 Gy and spinal radiation was commenced. During spinal radiation, repeat CT scanning revealed no intracranial lesion, so the attending radiotherapist decided not to give any further radiation to the brain. Patient no. 3 received radiation for a suspected germinoma, but the shrinkage of his tumour was not as quick as that of typical germinoma, so the residual mass was totally resected after 18.7 Gy of irradiation. The specimen contained a few germinoma cells along with granulomatous tissue, which is also characteristic of germinoma (Kraichoke *et al.*, 1988). Since the lesion had been resected totally, no further postoperative irradiation was given.

As a radiation sequela, brain necrosis developed in one patient (no. 1 in Table II) who received irradiation twice as described previously (Shibamoto *et al.*, 1988). One patient developed a chordoma within the irradiated volume at 5 years after therapy. The tumour was surgically removed and she is currently doing well. The intellectual status more than 1 year after radiotherapy was evaluable in 77 patients. We repeatedly asked the patients and their parents about their memory function. Excluding 14 patients who had functional defects that were probably due to the disease itself, evident recent memory disturbance developed in 2 of the 17 patients who had received doses of 54 Gy or more and in 2 of the 20 patients who had received $50 \pm 2$ Gy. In contrast, none of the 26 patients who received doses of 47 Gy or less developed memory disturbance. Evaluation of radiation-induced endocrine dysfunction was generally difficult because many patients had such symptoms as a result of the original disease, but anterior pituitary dysfunction developed after radiotherapy in two patients who received 60 Gy and 50 Gy, while it was not identified in the patients receiving lower doses.

### Discussion

A radiation dose of 50 Gy has been most commonly used to treat intracranial germinoma. The risk of normal brain tissue developing radiation necrosis after this dose is estimated to be less than 0.5%, and thus 50 Gy is generally regarded as a safe dose of radiation (Sheline *et al.*, 1980). However, even if brain necrosis does not occur, other adverse effects can develop, such as hypothalamic–pituitary dysfunction and a decrease in intellectual ability, including loss of recent memory (Duffner *et al.*, 1985). Therefore, it seems quite important to investigate the possibility of dose reduction.

There have been a few previous reports on the successful treatment of patients with intracranial germinoma using doses of 45 Gy or less (Sung *et al.*, 1978; Amendola *et al.*, 1984; Fields *et al.*, 1987; Jenkin *et al.*, 1990). However, the numbers have been small and the follow-up period insufficient in most reports, so that no firm conclusions could be drawn. It is noteworthy, however, that a few patients have been successfully treated with very low doses. The relatively old literature reports two patients who are alive at 25 years and 18 years after receiving 1,800 R and 2,950 R of radiation respectively (Simson *et al.*, 1968; Camins & Mount, 1974). Recently, Aydin *et al.* (1992) reported on a patient with biopsy-proven germinoma who died accidentally after receiving 16 Gy of irradiation. At autopsy, no viable tumour cells were found and the authors suggested that this disease could possibly be cured by much lower doses than those currently used. Also in our series, one patient who only received 19.5 Gy is still recurrence free at 15 years.

On the other hand, we found in-field local recurrence in one patient who had a poor general condition and received 40 Gy in 27 fractions given over 49 days. Because of the protracted delivery of the radiation, this did not correspond to the current practice of delivering 40 Gy in 20–24 fractions over 4–5 weeks. The literature also records many patients who developed local recurrence after doses of 30–60 Gy (Simson *et al.*, 1968; Sung *et al.*, 1978; Amendola *et al.*, 1984). Since the radiation procedure is not necessarily described in detail, it is generally unclear from these reports whether local recurrence was due to underdosage or not. Some patients may still develop local recurrence even after 50 Gy of irradiation and we had one patient who developed recurrence 9 years after receiving a dose of 60 Gy. In a recent review, Fuller *et al.* (1994) found no correlation between the radiation dose and disease-free survival or overall survival. Our results also indicated that the prognosis is not different between patients receiving 40–45 Gy and 50 Gy or more. However, the toxicity of these lower doses appears to be less than that of radiation doses $\geqslant 50$ Gy. Therefore, it seems reasonable for the standard radiation dose to be reduced to 40–45 Gy for germinomas less than 4 cm in diameter. The next challenge may be to investigate the possibility of further dose reduction.

With regard to prophylactic craniospinal irradiation, the optimum dose is still unclear. CSF dissemination occurred in one of our patients who had positive cytology and intraventricular dissemination at presentation and received 23.7 Gy to the craniospinal axis. On the other hand, seven patients who had positive CSF cytology and received 20 Gy or less to the craniospinal axis are currently doing well 2–15 years after therapy. Thus, we will continue to investigate whether 20 Gy is an appropriate dose or not.

Systemic chemotherapy using cisplatin and etoposide is presently being investigated for germinoma in some institutions (Patel *et al.*, 1992; Yoshida *et al.*, 1993). The rationale for performing chemotherapy is that radiation can have adverse effects on the normal brain while chemotherapy does not. However, it has become clear that chemotherapy is associated with an unacceptably high recurrence rate (Yoshida *et al.*, 1993; Shibamoto *et al.*, 1994) and the long-term toxicity (particularly on the genital organs) is still unclear. Our study showed that intracranial germinoma can be cured with lower radiation doses than have been standard, thus eliminating the major adverse effects of radiation in adolescents and adults. Therefore, chemotherapy (in combination with even lower dose irradiation) may only be worth consideration for children in whom 40 Gy of irradiation might still have unfavourable effects and for recurrent cases.

Regarding the treatment volume, we are using an individualised approach. According to the review by Brada & Rajan (1990), the incidence of spinal seeding in intracranial germinoma was 13% following brain irradiation alone. This rate may be too low to justify the routine use of craniospinal irradiation, but is also not negligible. We recently found that patients with positive CSF cytology have a higher risk of CSF dissemination (Shibamoto *et al.*, 1994) and we give prophylactic craniospinal irradiation to such patients as well as those with CSF dissemination. On the other hand, we have electively treated 11 patients without such findings with focal irradiation since 1985 and have found no recurrence so far in a median follow-up period of 45 months. Even if CSF dissemination should develop following focal irradiation with 40 Gy, it would be possible to give a second course of irradiation of the craniospinal axis to 20–24 Gy and of the site of recurrence to 40–45 Gy (provided that recurrence is outside the irradiated volume). Therefore, successful salvage of CSF dissemination might well be expected, with or without additional chemotherapy. We recommend focal irradiation with the doses described above for all patients with no CSF dissemination and negative cytology.

The authors wish to thank Dr M. Koishi for valuable help in collecting data.

### References

AMENDOLA, B.E., MCCLATCHEY, K. & AMENDOLA, M.A. (1984). Pineal region tumors: analysis of treatment results. *Int. J. Radiat. Oncol. Biol. Phys.*, **10**, 991–997.

AYDIN, F., GHATAK, N.R., RADIE-KEANE, K., KINARD, J. & LAND, S.D. (1992). The short-term effect of low-dose radiation on intracranial geminoma. A pathologic study. *Cancer*, **69**, 2322–2326.

BLOOM, H.J.G. (1983). Primary intracranial germ cell tumours. *Clin. Oncol.*, **2**, 233-257.

BRADA, M. & RAJAN, B. (1990). Spinal seeding in cranial germinoma. *Br. J. Cancer*, **61**, 339-340.

CAMINS, M.B. & MOUNT, L.A. (1974). Primary suprasellar atypical teratoma. *Brain*, **97**, 447-456.

DEARNALEY, D.P., A'HERN, R.P., WHITTAKER, S. & BLOOM, H.J.G. (1990). Pineal and CNS germ cell tumors: Royal Marsden Hospital experience 1962-1987. *Int. J. Radiat. Oncol. Biol. Phys.*, **18**, 773-781.

DUFFNER, P.K., COHEN, M.E., THOMAS, P.R.M. & LANSKY, S.B. (1985). The long-term effects of cranial irradiation on the central nervous system. *Cancer*, **56**, 1841-1846.

FIELDS, J.N., FULLING, K.H., THOMAS, P.R.M. & MARKS, J.E. (1987). Suprasellar germinoma: radiation therapy. *Radiology*, **164**, 247-249.

FULLER, B.G., KAPP, D.S. & COX, R. (1994). Radiation therapy of pineal region tumors: 25 new cases and a review of 208 previously reported cases. *Int. J. Radiat. Oncol. Biol. Phys.*, **28**, 229-245.

JENKIN, D., BERRY, M., CHAN, H., GREENBERG, M., HENDRICK, B., HOFFMAN, H., HUMPHREYS, R., SONLEY, M. & WEITZMAN, S. (1990). Pineal region germinonas in childhood. Treatment considerations. *Int. J. Radiat. Oncol. Biol. Phys.*, **18**, 541-545.

KRAICHOKE, S., COSGROVE, M. & CHANDRASOMA, P.T. (1988). Granulomatous inflammation in pineal germinoma. A cause of diagnostic failure at sterotaxic brain biopsy. *Am. J. Surg. Pathol.*, **12**, 655-660.

LINSTADT, D., WARA, W.M., EDWARDS, M.S.B., HUDGINS, R.J. & SHELINE, G. (1988). Radiotherapy of primary intracranial germinomas: the case against routine craniospinal irradiation. *Int. J. Radiat. Oncol. Biol. Phys.*, **15**, 291-297.

PATEL, S.R., BUCKNER, J.C., SMITHSON, W.A., SCHEITHAUER, B.W. & GROOVER, R.V. (1992). Cisplatin-based chemotherapy in primary central nervous system germ cell tumors. *J. Neuro-Oncol.*, **12**, 47-52.

SHELINE, G.E., WARA, W.M. & SMITH, V. (1980). Therapeutic irradiation and brain injury. *Int. J. Radiat. Oncol. Biol. Phys.*, **6**, 1215-1228.

SHIBAMOTO, Y., ABE, M., YAMASHITA, J., TAKAHASHI, M., HIRAOKA, M., ONO, K. & TSUTSUI, K. (1988). Treatment results of intracranial germinoma as a function of the irradiated volume. *Int. J. Radiat. Oncol. Biol. Phys.*, **15**, 285-290.

SHIBAMOTO, Y., ODA, Y., YAMASHITA, J., TAKAHASHI, M., KIKUCHI, H. & ABE, M. (1994). The role of cerebrospinal fluid cytology in radiotherapy planning for intracranial germinoma. *Int. J. Radiat. Oncol. Biol. Phys.*, **29**, in press.

SIMSON, L.R., LAMPE, I. & ABELL, M.R. (1968). Suprasellar germinomas. *Cancer*, **22**, 533-544.

SPIEGEL, A.M., DI CHIRO, G., GORDON, P., OMMAYA, A.K., KOLINS, J. & POMEROY, T.C. (1976). Diagnosis of radiosensitive hypothalamic tumors without craniotomy. Endocrine and neuroradiological studies of intracranial atypical teratomas. *Ann. Int. Med.*, **85**, 290-293.

SUNG, D., HARISIADIS, L. & CHANG, C.H. (1978). Midline pineal tumors and suprasellar germinomas: highly curable by irradiation. *Radiology*, **128**, 745-751.

THOMAS, G.M. & WILLIAMS, S.D. (1992). Testis. In *Principles and Practice of Radiation Oncology*, 2nd ed. Perez, C.A. & Brady, L.W. (eds) pp. 1117-1130. J.B. Lippincott: Philadelphia.

WARA, W.M., JENKIN, R.D.T., EVANS, A., ERTEL, I., HITTLE, R., ORTEGA, J., WILSON, C.B. & HAMMOND, D. (1979). Tumors of the pineal and suprasellar region: Children's Cancer Study Group treatment results 1960-1975. A report from the Children's Cancer Study Group. *Cancer*, **43**, 698-701.

YOSHIDA, J., SUGITA, K., KOBAYASHI, T., TAKAKURA, K., SHITARA, N., MATSUTANI, M., TANAKA, R., NAGAI, H., YAMADA, H., YAMASHITA, J., ODA, Y., HAYAKAWA, T. & USHIO, Y. (1993). Prognosis of intracranial germ cell tumours: effectiveness of chemotherapy with cisplatin and etoposide (CDDP and VP-16). *Acta Neurochir.*, **120**, 111-117.